# SimpleKANSleepNet: a Kolmogorov–Arnold network based sleep stage classification method

**DOI:** 10.3389/fbinf.2026.1738132

**Published:** 2026-02-18

**Authors:** Xiaopeng Ji, Lei Wang, Yong Zhou

**Affiliations:** School of Computer Science and Technology/School of Artificial Intelligence, China University of Mining and Technology, Xuzhou, China

**Keywords:** artificial intelligence (AI), deep learning, electroencephalography (EEG), Kolmogorov–Arnold network, sleep stage classification

## Abstract

A novel Kolmogorov–Arnold Network (KAN) based machine learning model is proposed for the automatic sleep stage classification task. The redefined architecture of the Multilayer Perceptron (MLP) aims to build a more flexible model by using learnable activation functions. In this study, an effective KAN model named SimpleKANSleepNet is evaluated on two different datasets with temporal features and frequency features extracted from electroencephalography (EEG), electromyogram (EMG), electrooculogram (EOG), and electrocardiogram (ECG) signals through a dual-stream convolutional neural network (CNN). Compared with existing CNN-based methods and graph convolutional networks (GCNs), the proposed model achieves an overall classification accuracy, F1-score, and Cohen’s kappa on the ISRUC-S1 and the Sleep-EDF-153 datasets of 0.812, 0.793, 0.757, 0.928, 0.929, and 0.910, respectively, which demonstrates its competitive classification performance and generality. Moreover, several data balancing methods are tested on Sleep-EDF-153 to further evaluate the potential for achieving the best results. Finally, the factors that may affect the classification ability are tested on the ISRUC-S1 dataset.

## 1 Introduction

Sleep is a complex, cyclic physiological process paramount to human cognitive function, memory consolidation, and overall physical health (Siegel, 2005). Common sleep problems are widespread and pose serious health risks, including heart disease, weight problems, and reduced daily productivity (Zhang et al., 2024). Polysomnography (PSG) analysis is the most effective way to evaluate sleep quality and identify sleep disorders, which involves the simultaneous recording of multiple physiological signals. Traditionally, PSGs used for sleep analysis include electroencephalography (EEG), electrooculography (EOG), electromyography (EMG), and electrocardiography (ECG). Collected PSGs are segmented into 30-s epochs and categorized into five stages: Wake, N1, N2, N3, and REM (rapid eye movement), according to the American Academy of Sleep Medicine (AASM) guidelines (Berry et al., 2012). Initially, the sleep stage classification task is processed manually by trained sleep technologists. However, this scoring process is time-consuming, subjective, and susceptible to inter-scorer variability, which creates a critical bottleneck in sleep clinics and motivates the long-standing pursuit of reliable automated systems.

Machine learning approaches have been applied to automatic sleep stage classification tasks for several years, and many models have been proposed. Shallow classifiers or traditional machine learning methods, including Support Vector Machines (SVMs) (Hussein et al., 2023), Random Forests (Memar and Faradji, 2018), and decision trees (Alshammari, 2024), etc., have demonstrated initial success. However, feature engineering is an inevitable step before inputting data into these models. Extensive features are extracted from time, frequency, and time-frequency domains by domain experts manually, and their performance is heavily affected by the quality and comprehensiveness of the engineered features. The success of deep learning in classification tasks has propelled the evolution of automated sleep staging from rule-based heuristics and traditional machine learning to contemporary deep learning paradigms. These paradigms enable end-to-end learning from raw or minimally processed signals. Specifically, Convolutional Neural Networks (CNNs) are widely adopted to extract temporal features. Furthermore, bidirectional Long Short-Term Memory (Bi-LSTM) modules are often incorporated to model the temporal context and transition rules between successive epochs. More recently, Graph Convolutional Networks (GCNs) have been employed to explicitly learn the non-Euclidean, functional relationships between different brain regions, achieving state-of-the-art performance. Despite their impressive accuracy, these deep learning models are not without limitations. An inevitable drawback of these deep learning models is their “black-box” nature, which offers little insight into the decision-making process. This raises concerns about trustworthiness and clinical adoption, as understanding the reason behind a diagnosis is as crucial as the diagnosis itself.

Inspired by the Kolmogorov–Arnold representation theorem, a novel architecture called Kolmogorov–Arnold Network (KAN) was proposed recently, which represents a significant departure from traditional neural network architectures (Liu et al., 2025). Given its superior approximation capabilities compared to conventional multilayer perceptrons, KANs are advantageous in learning bio-signals across temporal dimensions.

In this work, we adapt and extend the KAN algorithm into a model named SimpleKANSleepNet for sleep stage classification. Our work is primarily motivated by two persistent challenges in current deep learning-based sleep staging: (1) the “black-box” nature of models like CNNs and GCNs, which hampers clinical trust and adoption due to a lack of interpretability; and (2) the potential limitations of fixed-activation MLPs in modeling the intricate, non-linear, and structured relationships inherent in multi-modal bio-signals. We hypothesize that KANs, with their superior function approximation efficiency through learnable activation functions on edges and their inherent potential for interpretability, are particularly well-suited to address these challenges. The main contributions of this study are:

•A novel hybrid architecture named SimpleKANSleepNet is proposed for the sleep stage classification task. It innovatively integrates a KAN-based classifier with a dual-stream CNN feature extractor and a Gate Recurrent Unit (GRU)-based temporal context module. This represents the first exploration of KANs in the domain of multi-modal sleep stage classification.
•We explore the potential of the KAN architecture to serve as an alternative to MLPs for modeling complex bio-signal relationships. This exploration aims to lay the groundwork for future research into more transparent sleep staging models, moving beyond a mere application study. This also allows a preliminary assessment of KAN’s potential for providing insights into model decisions, despite the interpretability constraints of our hybrid architecture.
•Five-state sleep classification experiments are conducted on the ISRUC-S1 (https://sleeptight.isr.uc.pt/) and the SleepEDF-153 (www.physionet.org/content/sleep-edfx/1.0.0/) (Kemp et al., 2000; Goldberger et al., 2000) datasets to evaluate classification performance and generality. The proposed model achieves an overall accuracy, F1-score, and Cohen’s kappa of 0.812, 0.793, 0.757, 0.928, 0.929, and 0.910, respectively. Several additional experiments are conducted on the ISRUC datasets to identify factors affecting the classification performance, including the number of signal channels, channel types, input feature domains, and the size of the dataset.

## 2 Related work

### 2.1 Sleep stage classification

Sleep stage classification is a crucial step in sleep analysis, where many automated methods have been reported with high performance. Deep learning approaches, especially deep learning models, have undergone a rapid evolution, progressively moving from single-modality data to multi-modal methods.

CNNs are the first deep learning models applied to the sleep stage classification task, which demonstrated an unparalleled ability to automatically learn discriminative features from raw bio-signals and eliminate the need for manual feature engineering. A significant milestone in the use of CNN models in sleep scoring is DeepSleepNet, which introduced a dual-path architecture: one path with large filters to capture broad frequency information and another with small filters to extract temporal details (Supratak et al., 2017). Furthermore, it incorporated a bidirectional Long Short-Term Memory (Bi-LSTM) layer following the CNN, to explicitly model the temporal transition rules between adjacent epochs. 2D-CNNs have also been applied to classify sleep stages, normally using frequency domain features. A hybrid model named feature fusion temporal convolutional network (FFTCN) is proposed by combining a 1D-CNN branch with a 2D-CNN branch (Bao et al., 2024). Raw signals are transformed from the time domain to the frequency domain through continuous wavelet transform (CWT), converting a 1D temporal signal into a 2D image for input. The temporal features are extracted from a 1D-CNN branch and finally concatenated as fusion features for classification. To capture the complex structure of multi-channel sleep data without relying on hand-crafted 2D representations, 3D-CNNs like the 3DSleepNet model are also proposed (Ji et al., 2023b). The 3D convolutional layers in 3DSleepNet can aggregate information from neighboring time points, different frequency bands, and across various channels simultaneously, offering a more integrated and holistic feature learning approach.

Although CNNs excel at processing Euclidean data, they often fail to capture the functional and spatial relationships among different channels. This gap motivated the adoption of Graph Convolutional Networks (GCNs), which are specifically designed to operate on non-Euclidean graph structures. GraphSleepNet (Jia et al., 2020b), Jumping Knowledge based Spatial-Temporal GCN (JK-STGCN) (Ji et al., 2022), and Multi-View Spatial-Temporal Graph Convolutional Network (MSTGCN) (Jia et al., 2021a) are three GCN models that use dynamic sleep graph to extract spatial features and learn relationships among channels. Their experimental results show that explicitly modeling inter-channel relationships can boost classification performance, and more and more GCN-related models are being proposed in the bio-signal processing field (Huang et al., 2025).

### 2.2 Kolmogorov–arnold network

**FIGURE 1:**
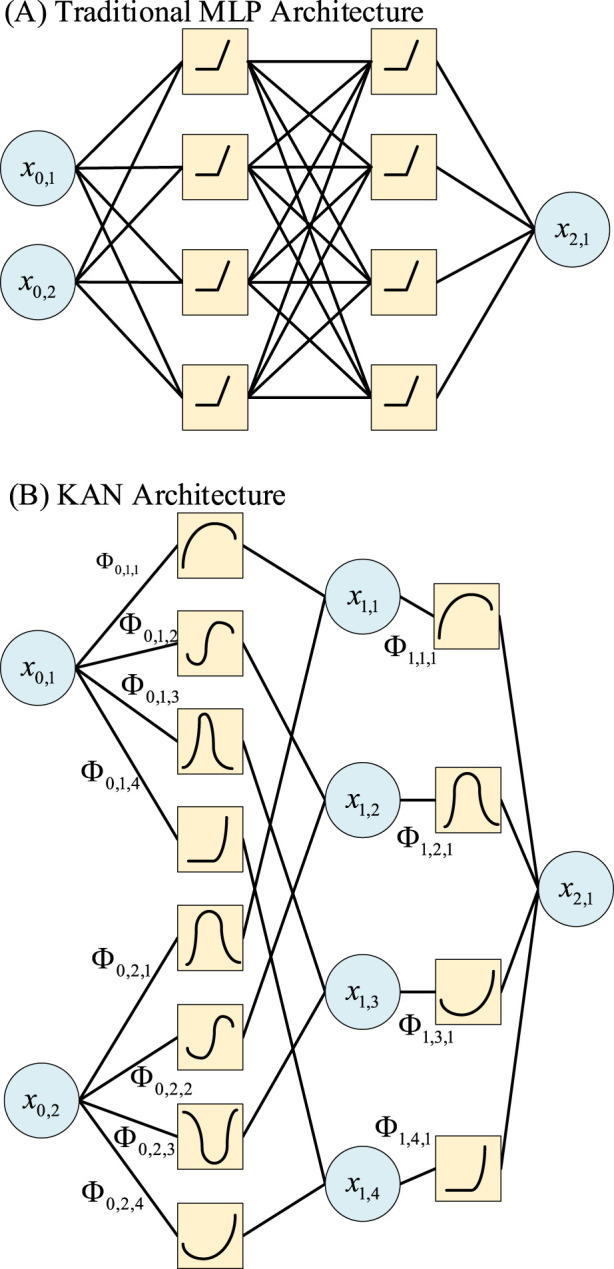
The comparison between a MLP and a KAN. **(A)** The MLP with learnable weights on nodes and fixed activation function. **(B)** A KAN with learnable activation function.

Kolmogorov–Arnold Networks (KANs) are a novel architecture designed to replace Multi-Layer Perceptrons (MLPs). Inspired by the Kolmogorov–Arnold representation theorem, which states that any multivariate continuous function can be expressed as a finite composition of continuous functions of a single variable (Liu et al., 2025). As Figure 1 shows, KANs place learnable univariate functions on the connections between nodes, rather than fixed non-linear activation functions on the nodes. This architecture allows learnable activation functions on the edges to adapt to complex data patterns more flexibly than fixed functions. KANs have been applied in many fields, such as traffic flow optimization (Zhang et al., 2025), medical image segmentation and generation (Li et al., 2025), and ECG analysis (Aghaomidi and Wang, 2024).

The physiological patterns of sleep stages are expressed through complex, non-linear dynamics in multi-channel biosignals such as EEG, EOG, EMG, and ECG. These signals exhibit intricate internal and mutual relationships. Traditional CNNs, GCNs, and MLPs, which rely on fixed nonlinearities like ReLU, may not represent these relationships optimally. In contrast, KANs are grounded in the Kolmogorov–Arnold Representation Theorem. This theorem states that any multivariate continuous function can be decomposed into a finite composition of univariate continuous functions. As a result, KANs can dynamically adapt their nonlinear transformations to fit specific patterns in the data. This adaptability may promote more efficient parameter use and provide a superior approximation of the complex mapping from multimodal features to sleep stages. Therefore, the inherent properties of KANs align well with the complexities of sleep signal analysis. Using a KAN as the core classifier could enable more flexible and powerful learning of the rules underlying sleep stage transitions from multimodal features.

## 3 Materials and methods

**FIGURE 2:**
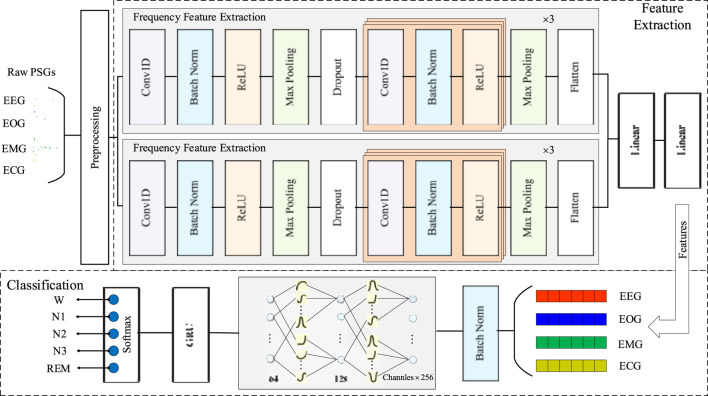
The architecture of the proposed model.

Figure 2 illustrates the architecture of the proposed SimpleKANSleepNet, which consists of three key stages: preprocessing, feature extraction, and classification.

### 3.1 Dataset and processing

1. ISRUC: The ISRUC dataset consists of three subsets, namely, ISRUC-S1, ISRUC-S2, and ISRUC-S3 (Khalighi et al., 2016). The S1 subset contains 100 adult subjects who showed evidence of sleep disorders, and each subject underwent a complete PSG recording. There are eight adult subjects with evidence of breathing sleep disorders, including two different recording sessions for each subject. All ten subjects from S3 are healthy individuals with no known sleep pathologies, and each subject has a complete PSG recording. All three subsets are labelled by two sleep experts according to the AASM standards. The detailed information about the ISRUC database is listed in Table 1. The PSGs are preprocessed by the data provider, where all EEG channels and EOG channels are filtered by a Butterworth filter with a frequency range of 0.3 Hz–35 Hz, while EMGs are filtered by a Butterworth filter with a range from 10 Hz to 70 Hz. The 50 Hz electrical noise is eliminated by a notch filter for all PSGs. Considering the dataset size and the subjects’ health conditions, only 50 random subjects are selected from ISRUC-S1 to conduct a 25-fold cross-validation for classification performance comparison and explore the factors that may affect the performance of the proposed model. To fully utilize the signal information, we select as many channels as possible. Consequently, all six EEG channels, two EOGs, one ECG, the Chin EMG, and the Leg-1 EMG are selected. All data used in our experiments are normalized epoch-by-epoch through z-score normalization:

$$X_{norm}^{i}=\frac{X^{i}-\mu }{\sigma },$$ (1)

where $X_{norm}^{i}$ is the normalized signal of the i -th sleep epoch, $X^{i}$ is the original signal of the i -th sleep epoch, μ and σ are the mean value and standard deviation of this epoch.

**TABLE 1:** Details about the ISRUC database.

Subset	Subjects	Age (years)	Sleep disorder	EEGs	EOGs	ECG	EMGs
Subgroup I	55 male, 45 female	51 ± 16	Yes	F3-A2, C3-A2 O1-A2, F4-A1 C4-A1, O2-A1	LOC-A2 ROC-A1	X2	Chin EMG (X1) Leg-1 EMG (X3) Leg-2 EMG (X4)
Subgroup II	6 male, 2 female	46.8 ± 18.8	Yes				
Subgroup III	9 male, 1 female	40 ± 10	No				

2. SleepEDF-153: The SleepEDF-153 dataset is part of the Sleep-EDF database and includes 78 subjects aged 25 to 101. Each subject had two sleep records, except for subjects 13, 36, and 52, from whom one night of data was lost due to a failing cassette, resulting in 153 available recordings. For each recording in the dataset, two EEG channels (Fpz-Cz and Pz-Oz) and one horizontal EOG channel, sampled at 100 Hz, are selected. The 50 Hz electrical noise was eliminated by a notch filter for all selected PSGs, followed by a wavelet transformation to remove noise. Finally, z-score normalization using Equation 1 was applied to normalize the preprocessed data for the feature extraction step.

### 3.2 Feature extraction

Signals of N channels are defined as $S=\left(s\left[1\right],s\left[2\right],\ldots s\left[L\right]\right)\in R^{L\times N\times T_{s}}$ , where L denotes number of sleep epochs and $T^{s}$ denotes number of data points in each epoch. For the l -th epoch $s\left[l\right]\in S\left(l\in 1,2,\ldots ,L\right)$ , the raw data of the i -th channel can be defined as $s^{i}\left[l\right]\in R^{T_{s}}\left(i\in \left\{1,2,\ldots ,N\right\},l\in \left\{1,2,\ldots ,L\right\}\right)$ . In the feature extraction stage, a dual-stream CNN is used as the feature extractor. For each CNN branch, 1D convolutional layers are used along the temporal dimension to aggregate temporal information for each input channel. The dual-stream architecture is employed because the branch with larger filters can capture broader frequency information, while the branch with smaller filters can better extract fine-grained temporal information. The output of the feature extractor is defined in Equation 2:

$$X^{i}=X_{S}^{i}\|X_{L}^{i},$$ (2)

where $X_{S}^{i}$ and $X_{L}^{i}$ are the outputs of the branch with smaller filters and larger filters, respectively, and ‖ is the concatenation operation.

The advantages of using a separate CNN feature extractor are:

1. The architecture of KANs is very similar to that of MLPs, and a large input size requires substantial computing resources. Therefore, feature extraction is an essential step in the proposed model to reduce the input dimensionality.
2. Moreover, KANs currently work only on the CPU. An integrated model that combines feature extraction and classification cannot utilize GPU acceleration. Hence, a separate feature extraction step is needed to accelerate the computation.
3. The CNN feature extractor ensures that both high-level temporal features and frequency features are captured without any prior knowledge or feature engineering, which are crucial for sleep stage classification tasks.

### 3.3 KANs

The Kolmogorov–Arnold Network (KAN) is a novel neural architecture fundamentally different from traditional Multi-Layer Perceptrons (MLPs). The idea of KANs is from the Kolmogorov–Arnold Representation Theorem, which states that any multivariate continuous function $f\left(x\right)=f\left(x_{1},x_{2},\ldots ,x_{n}\right)$ can be represented as a finite composition of continuous univariate functions like Equation 3 defined below:

$$f\left(x\right)=\sum_{q=1}^{2n+1}\Phi_{q}\left(\sum_{p=1}^{n}\phi_{q,p}\left(x_{p}\right)\right)$$ (3)

where $\phi_{q,p}:\left[0,1\right]\to R$ and $\Phi_{q}:R\to R$ are univariate functions. This theorem suggests that complex multivariate transformations can be broken down into a sum of simpler, univariate transformations.

Let $x_{0}\in R^{n_{0}}$ be the input vector where $n_{i}$ is the number of nodes in the i -th layer of the computational graph. A general KAN network is defined as Equation 4:

$$KAN\left(x\right)=\left(\Phi_{L-1}\circ \Phi_{L-2}\circ \cdots \circ \Phi_{1}\;\circ \;\Phi_{0}\right)x,$$ (4)

where $\Phi_{l}\left(l\in \left\{0,1,\right.\ldots ,L-\left.1\right\}\right)$ is the function matrix corresponding to the l -th KAN layer defined by Equation 5:

$$x_{l+1}=\underset{\Phi_{l}}{\underbrace{\left\{\begin{array}{cccc} \phi_{l,1,1}\left(\cdot \right) & \phi_{l,1,2}\left(\cdot \right) & \cdots & \phi_{l,1,n_{l}}\left(\cdot \right)\\ \phi_{l,2,1}\left(\cdot \right) & \phi_{l,2,2}\left(\cdot \right) & \cdots & \phi_{l,2,n_{l}}\left(\cdot \right)\\ \vdots & \vdots & & \vdots \\ \phi_{l,n_{l+!},1}\left(\cdot \right) & \phi_{l,n_{l+!},2}\left(\cdot \right) & \cdots & \phi_{l,n_{l+1},n_{l}}\left(\cdot \right) \end{array}\right\}}}x_{l},$$ (5)

where $\phi_{l,j,i}$ is the activation function connects $\left(l,i\right)$ and $\left(l+1,j\right)$ , which are the i -th neuron in the l -th layer and the j neuron in the ( l +1)-th layer, respectively.

Activation functions $\phi_{q,p}\left(x\right)$ in the KAN layer are residual activation functions defined by Equation 6:

$$\phi \left(x\right)=w_{b}b\left(x\right)+w_{s}\text{spline}\left(x\right),$$ (6)

where $w_{b}$ and $w_{s}$ are redundant but still included items. $b\left(x\right)$ is the silu function defined by Equation 7:

$$b\left(x\right)=\frac{x}{1+e^{-x}},$$ (7)

$\text{spline}\left(x\right)$ is parametrized as a linear combination of B-splines defined in Equation 8:

$$\text{spline}\left(x\right)=\sum_{i}c_{i}B_{i}\left(x\right),$$ (8)

where $c_{i}$ are the learnable coefficients for the B-spline basis functions $B_{i}\left(x\right)$ .

### 3.4 Gated Recurrent Unit (GRU)

Sleep is a dynamic process where the temporal context and transition rules between consecutive epochs are crucial for accurate staging. To capture these temporal dependencies, a Gated Recurrent Unit (GRU) is incorporated after the KAN-based feature fusion. GRU is a type of Recurrent Neural Network (RNN) that uses gating mechanisms to control the flow of information, effectively mitigating the vanishing gradient problem common in simple RNNs (Cho et al., 2014).

**FIGURE 3:**
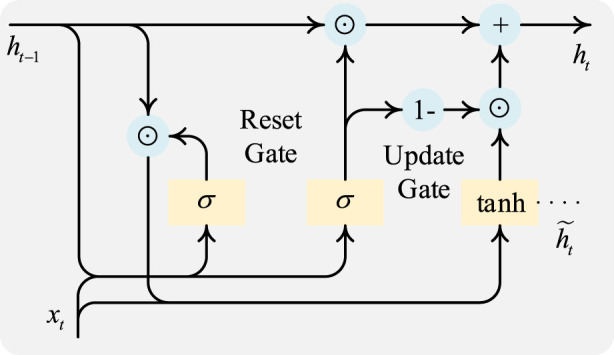
A GRU cell.

As Figure 3 shows, the GRU cell, at each time step t , takes the current input $x_{t}$ (the fused feature vector from our KAN module) and the previous hidden state $h_{t-1}$ , and produces a new hidden state $h_{t}$ . This hidden state serves as a compressed memory of the sequence up to time t . The internal computations of a GRU cell are governed by two gates: the reset gate $r_{t}$ and the update gate $z_{t}$ .

The formulas for the reset gate, the update gate, the candidate hidden state, and the final hidden state in a GRU cell are defined in Equations 9–12, respectively.

1. Reset Gate: This gate determines how much of the past hidden state $h_{t-1}$ should be “forgotten” or reset when computing the candidate activation.
$$r_{t}=\sigma \left(W_{xr}x_{t}+W_{hr}h_{t-1}+b_{r}\right)$$ (9)
2. Update Gate: This gate controls how much of the new hidden state $h_{t}$ will be composed of the candidate activation versus a copy of the old hidden state $h_{t-1}$ .
$$z_{t}=\sigma \left(W_{xz}x_{t}+W_{hz}h_{t-1}+b_{z}\right)$$ (10)
3. Candidate Hidden State: A candidate for the new hidden state is computed using the current input and the reset past hidden state.
$$\tilde{h_{t}}=\tan h\left(W_{xh}x_{t}+W_{hh}\left(r_{t}⊙h_{t-1}\right)+b_{h}\right)$$ (11)
  Here, ⊙ denotes the element-wise product. The reset gate $r_{t}$ allows the unit to drop information that is irrelevant in the future.
4. Final Hidden State: The final hidden state is a linear interpolation between the previous hidden state and the candidate hidden state, controlled by the update gate $z_{t}$ .

$$h_{t}=\left(1-z_{t}\right)⊙h_{t-1}+z_{t}⊙\tilde{h_{t}}$$ (12)

If the update gate $z_{t}$ is close to 1, the unit copies the candidate $\tilde{h_{t}}$ , effectively forgetting the past. If $z_{t}$ is close to 0, the unit retains most of the previous hidden state $h_{t-1}$ .

## 4 Experiments

### 4.1 Experiment setting

The classification performance evaluation experiments are conducted on the ISRUC-S1 and SleepEDF-153. For each experiment, we employed a subject-independent evaluation method to assess the model’s generalization capability. For a fair comparison between different models on the ISRUC-S1 subset, the experimental setting of the dataset remains consistent with the existing study. Hence, we randomly selected 50 subjects from the ISRUC-S1 dataset for a 25-fold cross-validation. In each fold, two subjects are randomly chosen as the validation set, and another two completely unseen subjects are held out as the test set. The remaining cases form the training set. To compare with other models fairly, we follow the same dataset dividing rule as the existing method in their experiments. A 10-fold cross-validation was performed on the SleepEDF-153 dataset. It is important to note that the SleepEDF-153 dataset consists of 153 sleep sessions (nights) from 78 subjects, as data from three nights are missing. Consequently, we treated each of these 153 sessions as an independent and distinct sample. This means that the two recording sessions from a single subject could potentially be allocated to different splits (e.g., one in the training set and the other in the validation or test set). However, sleep patterns and physiological signals from the same individual can vary significantly from one night to another due to factors such as changing sleep quality, mental state, and environmental conditions. Therefore, the data from the two nights are considered non-identical and statistically independent samples. For each fold, 13 or 14 random sessions were chosen as the validation set, and 15 or 16 unseen sessions were chosen as the test set.

Detailed hyperparameters of the CNN feature extractor and the proposed model are listed in Table 2. Moreover, the code will be uploaded to GitHub (https://github.com/ji-xiaopeng/SimpleKANSleepNet) once the paper is published.

**TABLE 2:** Hyperparameters of the feature extractor module and the KAN module.

Module	Hyperparameter		Value
Feature Extrator	Temporal branch	Conv 1D channel size	32–64–64–64
		Conv 1D kernel size	50-8-8-8
		Conv 1D stride size	6-1-1-1
		Max pooling kernel size	16–8
		Max pooling stride size	16–8
		Dropout rate	0.5
	Frequency branch	Conv 1D channel size	64–64–64–64
		Conv 1D kernel size	400-6-6-6
		Conv 1D stride size	50-1-1-1
		Max pooling kernel size	8–4
		Max pooling stride size	8–4
		Dropout rate	0.5
KAN	KAN layer		Channels * 256–128–64
	GRU		64–32

Overall evaluation metrics, including accuracy (ACC), F1-score, and precision (PR), are used to evaluate the overall performance, while per-class metrics, including precision (PR), recall (RE), and F1-score are also tested.

All experiments are carried out on a workstation with an Intel I7-10700 CPU, 96 GB memory, and a Nvidia GeForce RTX 2080 Ti GPU.

### 4.2 Comparison with the state-of-the-art methods

According to the comprehensive experimental results presented in Table 3, a comparative analysis reveals the distinct performance landscape of various deep learning models for sleep stage classification on the ISRUC-S1 subset. The proposed SimpleKANSleepNet demonstrates highly competitive performance, achieving an overall accuracy of 0.812 and an F1-score of 0.793, positioning it among the top-tier models. The detailed evaluation metrics on this dataset for each class are listed in Table 4.

**TABLE 3:** Comparison between the proposed model and other methods on the 50 random subjects from the ISRUC-S1 subset.

Method	Overall metrics			Per-class F1-score (F1)				
	ACC	F1	κ	W	N1	N2	N3	REM
DeepSleepNet	0.730	0.691	0.654	0.850	0.385	0.739	0.830	0.648
TinySleepNet	0.764	0.745	0.695	0.846	0.548	0.729	0.830	0.794
MMCNN	0.769	0.736	-	0.849	0.437	0.770	0.843	0.781
SeqSleepnet	0.770	0.683	-	0.844	0.124	0.769	0.853	0.794
GraphSleepNet	0.780	0.751	0.715	0.889	0.463	0.763	0.825	0.813
MSTGCN	0.808	0.787	0.752	0.885	0.539	**0.799**	**0.876**	0.838
StAGN	0.811	0.790	-	0.895	0.547	0.797	**0.876**	0.836
Metasleeplearner	0.710	0.678	-	0.772	0.442	0.680	0.802	0.697
Cosleep	0.579	0.501	-	-	-	-	-	-
FFTCN	0.774	0.745	0.71	0.865	0.450	0.767	0.863	0.779
SimpleKANSleepNet	**0.812**	**0.793**	**0.757**	**0.898**	**0.554**	0.796	0.863	**0.857**

^a^ W = awake. N1, N2 and N3 are sleep stage 1, 2, 3, separately, and are non-rapid eye movement. REM, rapid eye movement.

Bold numbers represent the best metric values, and underlined numbers represent the second best.

**TABLE 4:** Confusion matrix of the proposed model on the ISRUC-S1 dataset.

True labels	Predicted					Per-class metrics	
	W	N1	N2	N3	REM	PR	RE
W	9261	582	175	16	63	0.878	0.917
N1	866	2826	1480	29	354	0.606	0.509
N2	250	871	11059	839	231	0.760	0.835
N3	16	12	1396	7249	2	0.891	0.836
REM	146	374	437	3	4819	0.881	0.834

^a^ W = awake. N1, N2 and N3 are sleep stage 1, 2, 3, separately, and are non-rapid eye movement. REM, rapid eye movement; PR = precision; RE = recall.

Early end-to-end CNN models like DeepSleepNet (Supratak et al., 2017) and TinySleepNet (Supratak and Guo, 2020) established a strong baseline for sleep staging through deep learning methods. However, their overall and per-class performance is much lower than that of other models. This phenomenon may be due to the limited number of channels they use. While subsequent models like MMCNN (Chambon et al., 2018) and SeqSleepnet (Phan et al., 2019b) improved overall accuracy, SeqSleepNet’s exceptionally low N1 score (F1 = 0.124) highlights the difficulty of capturing its features with certain sequence modeling approaches.

Metasleeplearner (Banluesombatkul et al., 2021) and Cosleep (Ye et al., 2022) show the lowest performance among all models, whose frameworks are very special compared to other models. Metasleeplearner applies a simpler version of the DeepSleepNet architecture based on Model Agnostic Meta-Learning (MAML), while the Cosleep model is a co-training scheme, which exploits complementary information from multiple views. Given that the performance of the DeepSleepNet model is lower than expected, it is difficult for a simpler version to achieve an acceptable result. In terms of the Cosleep model, even though many effective modules are added, the self-supervised learning scheme still cannot achieve performance as high as that of supervised methods.

The introduction of graph-based models marked a significant advancement. GraphSleepNet (Jia et al., 2020a) demonstrated the benefit of modeling inter-channel relationships, notably improving the F1-score for the Wake stage. Its successors, MSTGCN (Jia et al., 2021a) and StAGN (Chen et al., 2023), further refined this paradigm by incorporating multi-view spatial-temporal convolutions and attention mechanisms, respectively, pushing the overall accuracy above 0.808.

Our proposed SimpleKANSleepNet achieves a balanced and robust performance. Its most notable strength lies in its best-in-class performance on the most challenging stages. It achieves the highest F1-score for the N1 stage (0.554) and the REM stage (0.857), which suggests that the Kolmogorov-Arnold Network’s capacity for efficient and highly non-linear function approximation is particularly effective at capturing the subtle and complex patterns that characterize these stages. The strong performance on Awake, N1, N2, and REM, coupled with high overall metrics, validates the KAN architecture as a powerful and promising alternative for biosignal processing.

The performance landscape on the Sleep-EDF-153 dataset reveals different model characteristics. Early CNN-based methods or temporal convolutional networks (TCNs), like the DeepSleepNet (Supratak et al., 2017), SleepPrintNet (Jia et al., 2020a), MultitaskCNN (Phan et al., 2019a), DeepResNet (Sun et al., 2018), SWTCNN (Jadhav and Mukhopadhyay, 2022), and FFTCN (Bao et al., 2024), consider signal types, channel sizes, or electrode locations only to construct models without any attention mechanism, which presents low but acceptable performance. Motivated by the performance enhancement from the attention mechanism, many sleep stage classification algorithms start to incorporate attention layers to help models capture important information from data. Models with attention layers, especially those using multiple PSG channels, can pay more attention to the most valuable information in sleep stage classification. As a result, models, like the SleepEEGNet (Mousavi et al., 2019), AttnSleepNet (Eldele et al., 2021), MultiChannelSleepNet (Dai et al., 2023), MaskSleepNet (Zhu et al., 2023), SalientSleepNet (Jia et al., 2021b), and MMASleepNet (Yubo et al., 2022) show improved results. On the other hand, MixSleepNet (Ji et al., 2023a) processes temporal features, frequency features, and time-frequency features by combining 3D-CNNs, GCNs, and 2D-CNNs, which enables this model to analyze PSGs from multiple domains, leading to a higher performance than other existing methods.

On this dataset, SimpleKANSleepNet establishes a new state-of-the-art by achieving the highest overall accuracy (0.907) and Cohen’s Kappa (0.807). This underscores its superior generalization capability and robust pattern recognition. Compared with the results on the ISRUC-S1 subset, a notable phenomenon of the results of the SimpleKANSleepNet on the Sleep-EDF-153 dataset is that the F1-score for the N1, N3, and REM stages decreases by 0.087, 0.164, and 0.057, respectively. One possible explanation is that the N1 stage is a transitional stage between the Awake stage and N2 stage, which has the characteristics of N1 and N2 at the same time, leading to the difficulty in classification. In automated sleep stage classification methods, N3 stages are sometimes misclassified into N2 stages. The boundaries between N2 and N3 sleep are blurred because the progression of slow-wave activity is a continuum, making the application of the arbitrary 20% threshold for classification inherently subjective. Moreover, the Awake stage ratio in the Sleep-EDF-153 dataset is much higher than that in the ISRUC-S1 subset, leading to a label-imbalanced problem. The lower overall F1-score and the higher overall Cohen’s kappa also indicate this issue. Due to the fact that most models compared in Table 5 use data augmentation techniques to enhance the performance, three data balance strategies are further tested and listed in Table 5 for a fair comparison between the proposed method and other methods. Meanwhile, the detailed classification metrics of a confusion matrix for each class are listed in Table 6.

**TABLE 5:** Comparison between the proposed model and other methods on the SleepEDF-153 dataset.

Method	Overall metrics			Per-class F1-score (F1)				
	ACC	F1	κ	W	N1	N2	N3	REM
DeepSleepNet	0.820	0.769	0.760	0.847	0.466	0.859	0.848	0.824
SleepPrintNet	0.816	0.765	0.747	0.927	0.474	0.836	0.800	0.788
MultitaskCNN	0.796	0.728	0.72	0.909	0.397	0.832	0.766	0.735
DeepResNet	0.820	-	0.769	0.847	0.466	0.859	0.848	0.824
SWTCNN	0.825	0.751	0.750	0.928	0.395	0.855	0.832	0.744
FFTCN	0.826	0.771	0.760	0.922	0.473	0.848	0.800	0.810
SleepEEGNet	0.800	0.736	0.730	0.917	0.441	0.825	0.735	0.761
AttnSleepNet	0.717	0.685	0.637	0.842	0.428	0.715	0.819	0.611
MultiChannelSleepNet	0.850	0.796	0.790	0.940	0.530	0.869	0.818	0.826
MaskSleepNet	0.838	0.742	0.830	0.879	0.506	0.897	0.860	0.709
SalientSleepNet	0.826	0.765	0.759	0.923	0.505	0.844	0.712	0.842
MMASleepNet	0.827	0.776	0.761	0.929	0.491	0.849	0.813	0.798
MixSleepNet	0.891	0.685	0.770	0.970	0.227	0.815	0.760	0.652
SimpleKANSleepNet (All data)	0.907	0.752	0.807	0.978	0.467	0.818	0.699	0.800
SimpleKANSleepNet (Sleep state focus)	0.833	0.771	0.769	0.932	0.525	0.849	0.699	0.851
SimpleKANSleepNet (Oversampling balance)	0.847	0.846	0.808	0.912	0.787	0.744	0.859	0.929
SimpleKANSleepNet (Undersampling balance)	**0.928**	**0.929**	**0.910**	**0.985**	**0.904**	**0.863**	**0.928**	**0.964**

^a^ W = awake. N1, N2 and N3 are sleep stage 1, 2, 3, separately, and are non-rapid eye movement. REM, rapid eye movement.

Bold numbers represent the best metric values, and underlined numbers represent the second best.

**TABLE 6:** Confusion matrix of the proposed model on the SleepEDF-153 dataset after sleep state focus balance.

True labels	Predicted					Per-class metrics	
	W	N1	N2	N3	REM	PR	RE
W	58875	2941	379	37	324	0.922	0.941
N1	3873	10589	5032	74	1693	0.555	0.498
N2	588	4442	59170	1661	2184	0.829	0.870
N3	26	37	4927	7891	18	0.816	0.612
REM	476	1059	1907	10	21830	0.838	0.864

^a^ W = awake. N1, N2 and N3 are sleep stage 1, 2, 3, separately, and are non-rapid eye movement. REM, rapid eye movement; PR = precision; RE = recall.

#### 4.2.1 Sleep state focus

The simplest data balance strategy is the sleep state focus, which retains only 30 min of wake periods before and after the sleep periods. This method helps the model focus on the sleep state rather than the Awake state. This Awake stage balancing process enhances N1, N2, and REM classification performance, while the N3 stage performance remains at a similar level due to its low proportion.

#### 4.2.2 Oversampling

The oversampling strategy generates new data based on existing data points, solving the data imbalance problem by increasing the number of samples in the minority class to match the number in the majority class. In our experiment, the oversampling method is applied after the sleep state focus strategy, which means that all sleep stages will be oversampled to the Awake stage of 1-h periods, patient by patient. The experimental results demonstrate that the minority classes, especially the N1 and N3 stages, increase significantly, with a slight decrease in the Awake and N2 stages.

#### 4.2.3 Undersampling

Unlike the oversampling strategy, the undersampling strategy will decrease the number of samples in the majority class to the number of samples in the minority class and reduce the total dataset size to balance the classes. However, the resampled dataset size will be extremely affected if the distribution of classes is extremely imbalanced. In our experiment, the undersampling strategy is applied directly to the preprocessed SleepEDF-153 dataset. The experimental results show that all performance metrics achieve the highest compared with other models. A possible explanation for this phenomenon is that the undersampling strategy will reduce data diversity, leading both the training set and testing set to a similar distribution if compared to the oversampling strategy.

**FIGURE 4:**
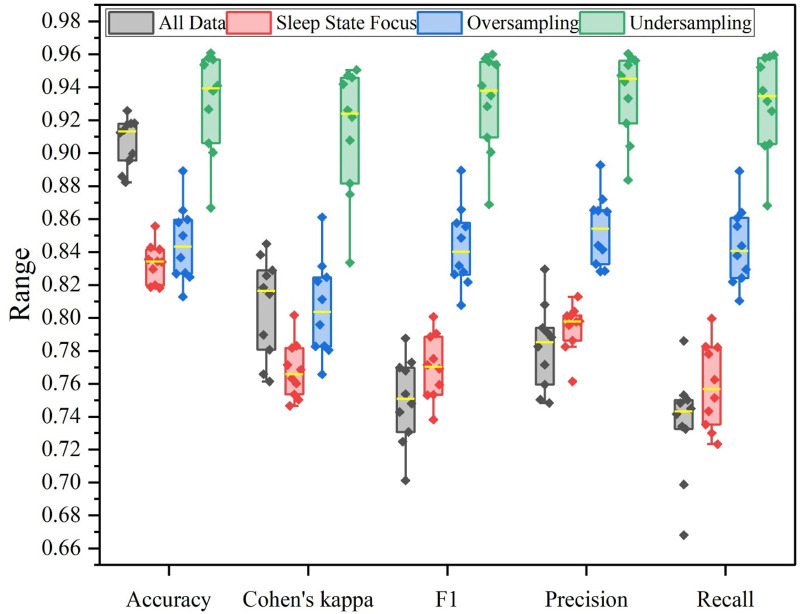
Box plot of four experimental settings on the Sleep-EDF 153 dataset.

To further evaluate the effectiveness and stability of three data augmentation techniques, a box plot is presented in Figure 4. For the situation of all data, the overall accuracy achieves the second-highest performance, while the lowest recall indicates that the model misclassifies data of the minority class into other labels. Obviously, its high classification accuracy is due to the imbalanced classes. Similarly, the sleep state focus balance strategy has little improvement on the recall or Cohen’s kappa metrics. The oversampling enhances the F1, precision, and recall metrics by balancing the class labels. The newly generated data points have a similar distribution to the existing data, which ensures the evaluation metrics are at the same level. As a result, it has the most stable classification performance on each fold. Undersampling consistently achieves the highest scores across all evaluation metrics, with performance values predominantly above 0.90. However, a few points are far from the mean value, which indicates lower stability than the oversampling strategy.

### 4.3 Model analysis

Several additional experiments are conducted on the ISRUC-S1 subset to further test the factors that may affect the classification performance and the evaluation results are listed in Table 7.

**TABLE 7:** Additional comparison experiments on the ISRUC-S1 Subset.

#	Channels	Subjects	Features	Overall metrics			Per-class F1-score (F1)				
				ACC	F1	κ	W	N1	N2	N3	REM
i	1 EEG, 1 ECG	50	CNN	0.721	0.684	0.640	0.824	0.410	0.728	0.824	0.635
ii	1 EEG, 1 EMG	50	CNN	0.761	0.735	0.693	0.861	0.458	0.747	0.836	0.770
iii	1 EEG, 1 EOG	50	CNN	0.770	0.749	0.704	0.882	0.515	0.750	0.831	0.765
iv	1 EEG, 1 EOG, 1 EMG, 1 ECG	50	CNN	0.774	0.752	0.709	0.868	0.485	0.761	0.839	0.806
v	2 EEG	50	CNN	0.759	0.732	0.690	0.868	0.489	0.749	0.846	0.709
vi	2 EOG	50	CNN	0.725	0.699	0.645	0.824	0.424	0.710	0.820	0.719
vii	6 EEG	50	CNN	0.775	0.751	0.710	0.871	0.500	0.770	0.844	0.771
viii	6 EEG, 2 EOG	50	CNN	0.782	0.759	0.719	0.875	0.510	0.774	0.845	0.790
ix	All channels	50	CNN	0.812	0.793	0.757	0.898	0.554	0.796	0.863	0.857
x	All channels	50	DE	0.753	0.710	0.679	0.831	0.358	0.749	0.810	0.802
xi	All channels	50	STFT	0.754	0.729	0.682	0.862	0.452	0.737	0.783	0.811
xii	All channels	50	Statistic	0.720	0.644	0.635	0.808	0.133	0.719	0.797	0.760
xiii	All channels	20	CNN	0.739	0.703	0.662	0.860	0.442	0.710	0.741	0.764

^a^ W = awake. N1, N2 and N3 are sleep stage 1, 2, 3, separately, and are non-rapid eye movement. REM, rapid eye movement.

A clear conclusion can be drawn from experiments i, ii, iii, iv, and v: ECG, EMG, and EOG contribute differently to the classification performance, among which EOG contributes the most, especially for the REM stage. This phenomenon can be explained by the fact that sleep stages are categorized into REM and non-REM, with the EOG signal being a significant characteristic for identification. Consequently, EOG helps to improve the overall performance and REM stage classification. Moreover, experiments v and vi further demonstrate the importance of EEG signals for sleep stage identification, while a high performance can also be achieved by increasing the number of EOG channels. Experiments v and vii demonstrate that increasing the number of EEG channels can enhance performance, but a greater improvement is achieved through multimodal data, as shown by experiments vii, viii, and ix.

The comparison between experiments ix, x, xi, and xii shows the necessity of the CNN feature extractor, where differential entropy (DE), Short-Time Fourier Transform (STFT), and statistical features represent frequency domain, time-frequency domain, and temporal domain features, respectively. A simple but important conclusion can be drawn from the comparison: the CNN feature extractor can extract high-level temporal and frequency features without any prior knowledge. Moreover, pure frequency features or pure temporal features fail to capture features from the other domain, which are important for identifying a sleep epoch. Although the STFT method can extract time-frequency domain features, time resolution and frequency resolution are inversely related. Therefore, the results obtained from STFT are lower than those from CNN features.

**FIGURE 5:**
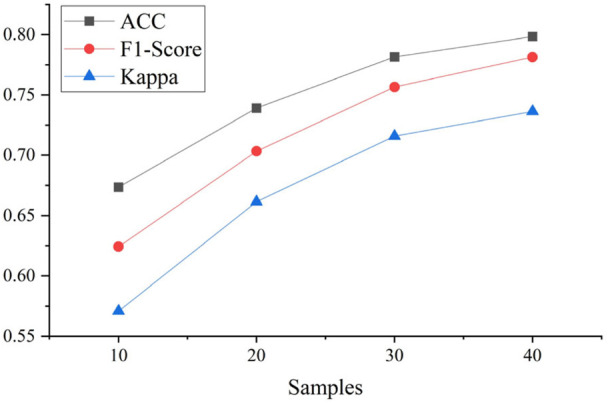
Performance increasing trend with samples.

The comparison between experiment xii and xiii shows the importance of sample size for classification performance and, Figure 5 shows the trend of performance improvement with increasing sample size. A fundamental observation is that the data size has a crucial impact on performance, and the proposed model can classify sleep stages correctly if sufficient data are available for training.

### 4.4 Ablation study

**FIGURE 6:**
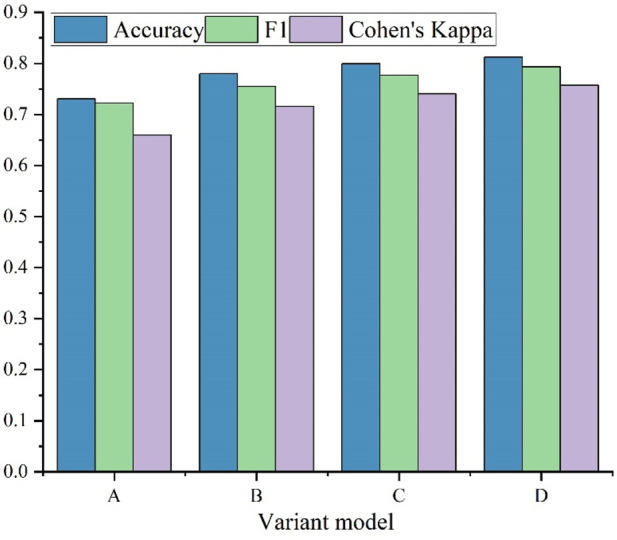
Comparison of the designed variant models.

To further evaluate the effectiveness of the KAN module and the GRU module, four variant models are designed to conduct ablation experiments on the ISRUC-S1 subset. Figure 6 illustrates the comparison among four variant models. The details of these models are:

Variant A: The simplest variant model, consisting of a CNN feature extractor and a GRU cell.

Variant B: The CNN is used to extract features, which are fed into a KAN module without a GRU cell.

Variant C: An MLP is used to replace the KAN module with a GRU cell after the CNN feature extractor.

Variant D: The proposed SimpleKANSleepNet model.

Variant A achieves the lowest performance among all models. A reasonable explanation is that the CNN module can extract features effectively, and the GRU cell can learn transitional rules efficiently as well. However, the built-in classification part consisting of two MLP layers is too shallow to learn features. Variant B has a slight performance improvement, but it is still unacceptable. Variant C further improves the experimental results, but they are still lower than the proposed model.

The comparison between variant A and variant C shows the necessity of an independent classifier. A key point here is that model C cannot be seen as simply adding MLP layers to model A. Since the training process of model A with deeper MLP layers is different from that of model C. For the model A with deeper MLP layers, the training process will update all weights in a training step, and it will consider the whole model. However, the extractor part will be frozen during the training of the independent MLP classifier.

The comparison between variant C and variant D shows the necessity of a KAN module. Variant C uses a fixed activation. The KAN module, with its learnable activation functions, is better suited to modeling the complex interdependencies in PSG data than the fixed-activation MLP used in Variant C. Hence, the proposed model achieves a better performance.

The comparison between variant B and variant D shows the necessity of a GRU cell to learn the transition rules among neighbouring sleep epochs.

## 5 Conclusion

In this study, a novel Kolmogorov–Arnold Network-based model, named SimpleKANSleepNet, is proposed to classify sleep stages. Temporal features and frequency features are extracted by a dual-branch CNN using large and small filters, respectively. These features are then fed into a three-layer KAN for classification. The experimental results on the ISRUC-S1 subset show that the overall accuracy, the F1-score, and Cohen’s kappa reach 0.812, 0.793, and 0.757, respectively, which achieves comparable performance. Experimental results on SleepEDF-153 show that the proposed model has a high performance on the class of majority samples, if the data is imbalanced. However, the classification performance for minority classes can be improved through data balancing strategies, especially with the undersampling strategy. It also indicates a drawback that the KAN model is sensitive to data distribution. Another drawback of the proposed model is the computational speed. Currently, the KAN-based model can only be trained on CPUs, while other deep learning architectures are GPU-based, which limits its architecture construction and execution efficiency. However, this characteristic also allows the deployment of artificial intelligence devices. Moreover, the proposed two-step model architecture splits the feature extraction and classification process, which also prevents the KAN from providing interpretability due to the non-interpretable features generated by CNN. In the future, one potential research direction is to develop a GPU-based KAN model, which would address both interpretability and computational speed issues. Furthermore, it is worthwhile to explore new KAN-based architectures for sleep stage classification tasks.

## Data Availability

The original contributions presented in the study are included in the article/supplementary material, further inquiries can be directed to the corresponding author.
